# Bayesian hypothesis testing and estimation under the marginalized random-effects meta-analysis model

**DOI:** 10.3758/s13423-021-01918-9

**Published:** 2021-06-22

**Authors:** Robbie C. M. van Aert, Joris Mulder

**Affiliations:** Department of Methodology and Statistics, Tilburg University, Tilburg, the Netherlands

**Keywords:** Bayes factor, Meta-analysis, Random-effects model, Heterogeneity

## Abstract

Meta-analysis methods are used to synthesize results of multiple studies on the same topic. The most frequently used statistical model in meta-analysis is the random-effects model containing parameters for the overall effect, between-study variance in primary study’s true effect size, and random effects for the study-specific effects. We propose Bayesian hypothesis testing and estimation methods using the marginalized random-effects meta-analysis (MAREMA) model where the study-specific true effects are regarded as nuisance parameters which are integrated out of the model. We propose using a flat prior distribution on the overall effect size in case of estimation and a proper unit information prior for the overall effect size in case of hypothesis testing. For the between-study variance (which can attain negative values under the MAREMA model), a proper uniform prior is placed on the proportion of total variance that can be attributed to between-study variability. Bayes factors are used for hypothesis testing that allow testing point and one-sided hypotheses. The proposed methodology has several attractive properties. First, the proposed MAREMA model encompasses models with a zero, negative, and positive between-study variance, which enables testing a zero between-study variance as it is not a boundary problem. Second, the methodology is suitable for default Bayesian meta-analyses as it requires no prior information about the unknown parameters. Third, the proposed Bayes factors can even be used in the extreme case when only two studies are available because Bayes factors are not based on large sample theory. We illustrate the developed methods by applying it to two meta-analyses and introduce easy-to-use software in the R package BFpack to compute the proposed Bayes factors.

## Introduction

The rapidly expanding scientific literature calls for methods to synthesize research such as a systematic review. Meta-analysis is an important part of a systematic review and refers to applying statistical methods to combine findings of different studies on the same topic. The first step of a meta-analysis is to obtain a standardized effect size (e.g., standardized mean difference or correlation coefficient) for each included study and these effect sizes are subsequently combined by means of meta-analysis methods to summarize the included studies. Meta-analysis is nowadays seen as the gold standard for research synthesis (Aguinis et al., 2011; Head et al., 2015) and is often used for policy making as its results are seen as best available evidence (Thompson & Sharp, 1999; Cordray & Morphy, 2009; Hedges & Olkin, 1985).

The random-effects model is the most commonly used statistical model in meta-analysis (Borenstein et al., 2010; 2009). In the random-effects model, each study is assumed to have an unknown true underlying effect size. The main parameter of interest in this model is the overall effect size of the studies included in the meta-analysis. However, estimation and statistical inferences for the between-study variance in the random-effects model is just as important (Higgins et al., 2009) because both parameters focus on distinct and relevant aspects of a meta-analysis. For example, the overall effect size in a meta-analysis on the efficacy of a psychological treatment refers to the average efficacy of the treatment across studies and the between-study variance quantifies the heterogeneity across studies.

The vast majority of meta-analyses uses frequentist analysis techniques for estimation and drawing statistical inferences. However, Bayesian meta-analysis methods have been proposed as well and have gained in popularity (Xu et al., 2008). Bayesian methods are especially well suited for analyzing meta-analytic data (Smith et al., 1995; Sutton & Abrams, 2001; Lunn et al., 2013; Turner & Higgins, 2019) because the multilevel structure of a random-effects meta-analysis can be straightforwardly taken into account. Moreover, estimation of the between-study variance is imprecise using frequentist meta-analysis methods in the common situation of a meta-analysis containing a small number of studies (Chung, Rabe-Hesketh, & Choi, 2013; Sidik & Jonkman, 2007; Kontopantelis, Springate, & Reeves, 2013). Bayesian meta-analysis methods are advantageous in this situation because (i) externally available information about the between-study variance can be incorporated in the prior distribution if available, and (ii) the methodology does not directly rely on large sample theory.

Meta-analysts generally want to estimate and conduct hypothesis tests for the parameters in the random-effects model. The vast majority of the literature on Bayesian meta-analysis methods has been focused on parameter estimation using either empirical Bayes or fully Bayesian estimation (e.g., (Turner et al., 2015; Rhodes et al., 2015; Normand, 1999; Lambert et al., 2002)). However, hypothesis testing using Bayes factors has also been proposed, which quantifies evidence of one model relative to another model (Kass & Raftery, 1995). (Berry, 1998) proposed a Bayes factor for testing the null hypothesis of no between-study variance in a meta-analysis of studies using 2x2 contingency tables. Rouder and Morey (2011) developed a Bayes factor to test the null hypothesis of no effect in a meta-analysis of studies using a two-independent groups design. (Scheibehenne et al., 2017) and (Gronau et al., 2017) proposed a Bayesian model averaging approach to compute an average Bayes factor for testing the null hypothesis of no effect. In this approach, an average Bayes factor is computed by averaging over posterior model probabilities obtained with the random-effects model and the equal-effect model (a.k.a. fixed-effect or common-effect model) where the study’s true effect sizes are assumed to be homogeneous.

The first contribution of our paper is that we propose, in contrast to existing Bayesian meta-analysis methods, to use a *marginalized* random-effects meta-analysis (MAREMA) model rather than the random-effects model. In this MAREMA model, the study-specific true effects are regarded as nuisance parameters and integrated out of the probability density function. The elimination of nuisance parameters via integration is common in integrated likelihood methods (e.g., Berger et al., (1999)), and has recently been extended to marginal random intercept models (Mulder and Fox, 2013; 2019), and marginal item response theory models (Fox et al., 2017).

The proposed MAREMA model encompasses three important meta-analysis models. First, the equal-effect model is included if the between-study variance is equal to zero. Second, the random-effects model is included when the between-study variance is positive, which implies that the differences between studies’ effect sizes cannot be fully explained by sampling error. Third, the random-effects model in case of a negative between-study variance is also included. A negative between-study variance indicates that the differences between studies’ effect sizes are smaller than expected based on sampling error. A negative between-study variance may yield relevant insights for meta-analysts because it may indicate that the assumption of independence of primary studies in a meta-analysis is violated or that the computation of the effect sizes of the primary studies is incorrect (Ioannidis et al., 2006).

Our proposed methodology is also distinctive from other Bayesian meta-analysis methods because we place a prior distribution on the proportion of total variance that can be attributed to between-study variance rather than directly on the between-study variance parameter. This proportion is known as *I*^2^ (Higgins & Thompson, 2002; Higgins et al., 2003) in the meta-analysis literature and it is frequently used to quantify the relative heterogeneity around the true effect size (Borenstein et al., 2017). An advantage of placing a prior on *I*^2^ is that it is a bounded parameter which enables us to place a proper (noninformative) uniform prior to compute Bayes factors (note that Bayes factors generally cannot be computed using improper priors while at the same time arbitrarily vague proper priors should also be avoided due to Bartlett’s phenomenon (Jeffreys, 1961; Lindley, 1957; Bartlett, 1957). Due to the uniformity of the prior, the proposed Bayes factors can be used for a default Bayesian meta-analysis without requiring external prior knowledge about the model parameters.

The proposed Bayes factors enable testing point and one-sided hypotheses. Examples of a point and one-sided hypothesis are testing whether the overall effect size in a meta-analysis equals zero (i.e., *H* : *μ* = 0) or is larger than zero (i.e., *H* : *μ* > 0). Moreover, the proposed Bayes factors also enable testing multiple hypotheses simultaneously (e.g., *H* : *μ* = 0 vs. *H* : *μ* > 0 vs. *H* : *μ* < 0). Another attractive property is that the quantification of relative evidence between hypotheses is exact even in the extreme case of only two studies in a meta-analysis as Bayes factors do not rely on large sample theory.

The outline of this paper is as follows. We continue by further introducing the MAREMA model and illustrate Bayesian estimation under the MAREMA model. Subsequently, we introduce Bayes factor testing under the MAREMA model and elaborate on the specification of the prior distributions. In Section “Bayesian hypothesis testing in examples”, the MAREMA model is used to compute Bayes factors for a meta-analysis on the efficacy of two treatments for post-traumatic stress disorder (PTSD) and a meta-analysis on the effect of a smartphone application and cessation support on long-term smoking abstinence. We conclude the paper with a discussion section.

## Marginalized random-effects meta-analysis model

The conventional random-effects meta-analysis model (Borenstein et al., 2009; Konstantopoulos & Hedges, 2019) assumes that *i* = 1, 2, ..., *k* independent effect sizes are extracted from studies on the same topic. The statistical model of the random-effects model can be written as

1 $$  \begin{aligned} y_{i} \sim N(\theta_{i}, {\sigma_{i}^{2}}) \\ \theta_{i} \sim N(\mu, \tau^{2}) \end{aligned} $$

where *y*_*i*_ is the observed effect size of the *i* th study, *𝜃*_*i*_ is the study-specific (unknown) true effect size, *μ* is the overall true effect size, *τ*^2^ is the between-study variance in true effect size, and ${\sigma _{i}^{2}}$ is the known sampling variance of the *i* th study, which is conventionally estimated in practice and then assumed to be known in the analysis. The random-effects model simplifies to an equal-effect model if *τ*^2^ = 0 because all studies then share a common true effect size. Note that other distributions for the random effects than the normal distribution have been proposed (e.g., Baker & Jackson, 2008; 2016; Lee & Thompson, 2008), but a normal distribution for the random effects remains to be used in almost any random-effects meta-analysis.

The study-specific true effects *𝜃*_*i*_ are generally treated as nuisance parameters in the random-effects model. We integrate these out of the random-effects model in Eq. 1 to obtain the MAREMA model, which is given by (see also Raudenbush & Bryk, 1985 for instance)

2 $$  y_{i} \sim N\left( \mu, {\sigma}_{i}^{2}+\tau^{2}\right). $$

Multiple estimators have been proposed for estimating the between-study variance *τ*^2^ (Veroniki et al., 2016; Langan et al., 2016). Estimates of *τ*^2^ cannot be compared across meta-analyses if these meta-analyses used different effect size measures. That is, a *τ*^2^ estimate in a meta-analysis of standardized mean differences cannot be compared to one of correlation coefficients, and this was one of the reasons to develop *I*^2^ that will be described next (Higgins & Thompson, 2002).

### Quantifying heterogeneity using *I*^2^

A commonly used way to quantify the relative heterogeneity in a meta-analysis is using *I*^2^ (Higgins & Thompson, 2002; Higgins et al., 2003),

3 $$ I^{2} = \frac{\tau^{2}}{\tau^{2} + \tilde{\sigma}^{2}} $$

where $\tilde {\sigma }^{2}$ is the typical within-study sampling variance that is computed with

4 $$ \tilde{\sigma}^{2} = \frac{(k-1) \sum 1/{\sigma_{i}^{2}}}{\left( \sum 1/{{\sigma}_{i}^{2}}\right)^{2} - \sum 1/{\sigma_{i}^{4}}}. $$

*I*^2^ has an intuitive interpretation because it is the proportion of total variance that can be attributed to between-study variance in true effect size. Note that *I*^2^ resembles the intraclass correlation coefficient (ICC) that is routinely reported in multilevel analysis. This ICC indicates the proportion of total variance that can be attributed to taking into account the dependence of observations within the level 2 units (e.g., (Hox et al., 2018)). However, a major difference between *I*^2^ and the ICC is that the total variance in the computation of *I*^2^ (i.e., $\tau ^{2}+\tilde {\sigma }^{2}$) is a function of the studies’ sample size whereas the ICC is not a function of the sample size of the level 2 units. Hence, *I*^2^ artificially increases if the sample size of the primary studies increases while *τ*^2^ remains constant (Rücker et al., 2008).

Next, we reparameterize the MAREMA model in Eq. 2 using *I*^2^. We replace *I*^2^ with the Greek letter *ρ* to make explicit that it is an unknown parameter and can attain negative values. The MAREMA model can then equivalently be written as

5 $$  y_{i} \sim N(\mu, {\sigma^{2}_{i}}+\tilde{\sigma}^{2} \rho/(1-\rho)). $$

where $\tilde {\sigma }^{2} \rho /(1-\rho )$ transforms *ρ* to its corresponding *τ*^2^. In Eq. 5, *ρ* must lie in the interval (*ρ*_*min*_,1) with

6 $$ \rho_{min} = \frac{-\sigma_{min}^{2}}{-\sigma^{2}_{min}+\tilde{\sigma}^{2}} $$

where $\sigma ^{2}_{min}$ is the smallest sampling variance of the studies included in the meta-analysis. *ρ*_*min*_ is the smallest possible value of the parameter *ρ* given the observed data and is always negative. Note that the special case *ρ* = 0 (i.e., the equal-effect model) does not lie on the boundary of the parameter space, which enables testing the hypothesis of homogeneous true effect size.

## Bayesian estimation under the MAREMA model

The MAREMA model can be estimated using flat priors if prior information is absent,

7 $$  \begin{aligned} \pi(\mu, \rho) & = \pi(\mu) \pi(\rho), \text{with} \\ \pi(\mu) & \propto 1 \\ \pi(\rho) & = U(\rho_{min}, 1) \end{aligned} $$

where *U* refers to the uniform distribution. Flat priors are used for estimation to minimize the impact of the priors on the results. We illustrate Bayesian estimation under the MAREMA model by analyzing data of two meta-analyses. The first meta-analysis is by Ho & Lee, (2012) on the efficacy of eye movement desensitization and reprocessing (EMDR) therapy versus exposure based cognitive behavior therapy (CBT) to treat PTSD. This meta-analysis consists of ten standardized mean differences (i.e., Hedges’ *g*) and a positive effect size indicates that EMDR therapy is more efficacious than CBT. The second meta-analysis is by Whittaker et al.,, (2019) on the difference between using a smartphone app for smoking cessation support and lower intensity support on long-term abstinence. Three studies are included in this meta-analysis and log risk ratio is the effect size measure of interest. A positive log risk ratio indicates that using a smartphone app for smoking cessation support yields more long-term abstinence than lower intensity support. These examples were selected to illustrate that the proposed methodology can be used for different effect size measures and meta-analyses with only a small number of primary studies, which is especially common in medical research (Rhodes et al., 2015; Turner et al., 2015).

A Gibbs sampler is proposed for Bayesian estimation under the MAREMA model (see Appendix A). We also analyzed the data of these meta-analyses with frequentist random-effects meta-analysis using the restricted maximum likelihood estimator (Raudenbush, 2009) for estimating *τ*^2^ as implemented in the R package metafor (Viechtbauer, 2010). R code of these analyses is available at https://osf.io/jcge7/.

**Fig. 1:**
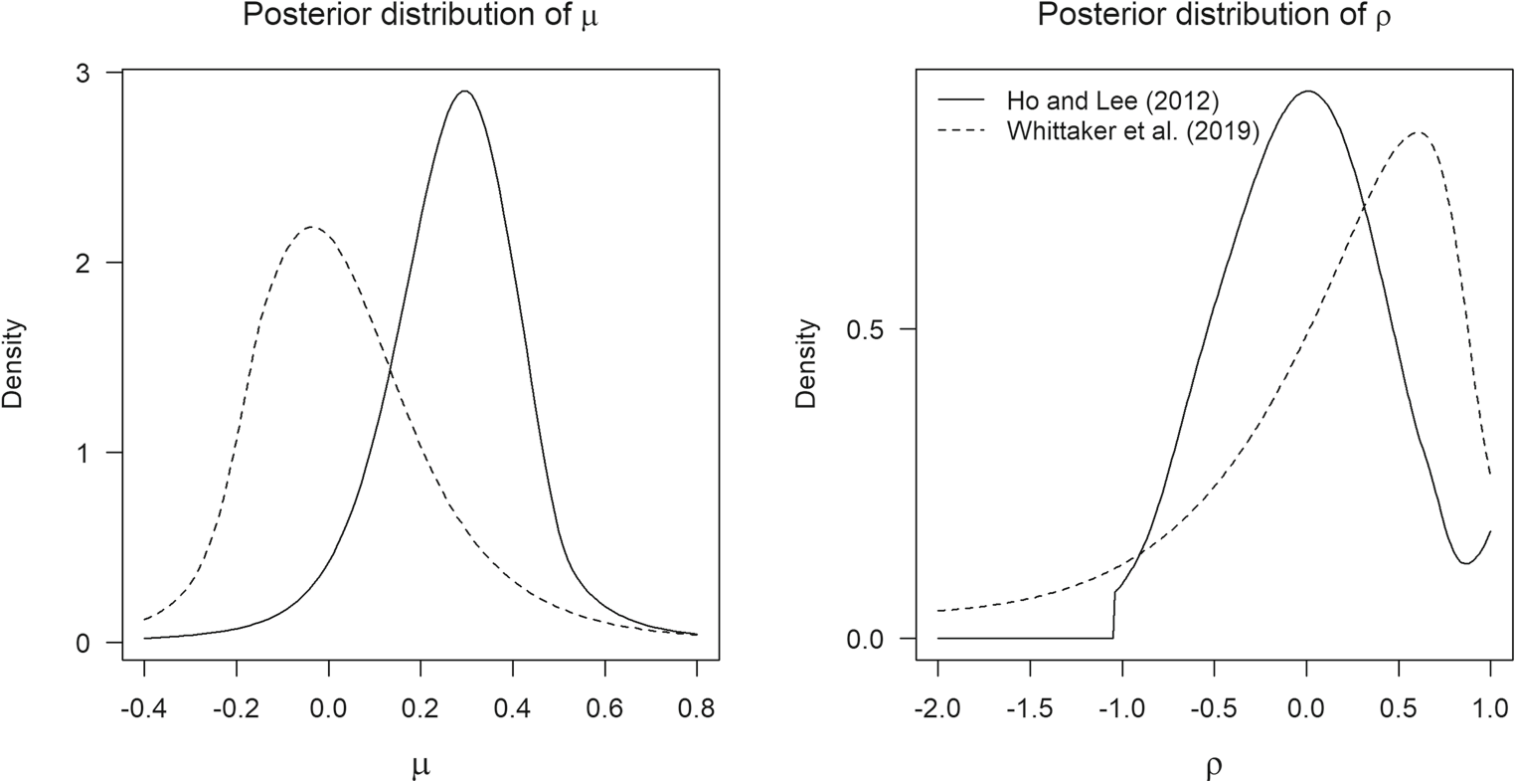
Posterior distributions of *μ* (*left panel*) and *ρ* (*right panel*) for the meta-analyses by Ho & Lee, (2012) (*solid lines*) and Whittaker et al.,, (2019) (*dashed lines*). The posterior distributions in the figure are smoothed using a logspline as implemented in the R package logspline (Kooperberg, 2020). *ρ*_*min*_ = − 1.045 for the meta-analysis by Ho & Lee, (2012) and *ρ*_*min*_ = − 2.326 for the meta-analysis by Whittaker et al.,, (2019)

The posterior distributions of *μ* and *ρ* when fitting the MAREMA model to the meta-analyses by Ho & Lee, (2012) (solid lines) and Whittaker et al.,, (2019) (dashed lines) are presented in Fig. 1. Remarkably, the posterior distributions of *ρ* (right panel of Fig. 1) are very wide for both meta-analyses. There is also considerable posterior support for negative *ρ* values. This could suggest that the random-effects model, which is employed for the frequentist analysis, may not be appropriate for these data. We will reflect on causes and the implications of a negative value for *ρ* in the discussion section.

Parameter estimates obtained with the MAREMA model and also the frequentist random-effects model are presented in Table 1. The first row of Table 1 shows the results of the MAREMA model and the second row those of the frequentist random-effects model. The first three columns show the results of estimating *μ* and the last three columns those of estimating *ρ*. The metafor package was used for the frequentist meta-analysis, which does not report a standard error for $\hat {\rho }$.

**Table 1:** Results of Bayesian estimation under the marginalized random-effects meta-analysis (MAREMA) model (using posterior means) and frequentist random-effects meta-analysis when estimating the parameters in the meta-analysis by Ho & Lee, (2012) (*first panel*) and Whittaker et al.,, (2019) (*second panel*)

	$\hat {\mu }$			$\hat {\rho }$		
	Estimate	SD/SE	95% CI	Estimate	SD/SE	95% CI
Ho & Lee, (2012)						
MAREMA	0.274 (0.327)	0.29	(-0.109;0.638)	–0.026 (-0.016)	0.425	(-0.837;0.812)
Frequentist	0.249	0.129	(-0.003;0.502)	0.022	−	(0;0.747)
Whittaker et al.,, (2019)						
MAREMA	0.033 (0.043)	0.381	(-0.413;0.625)	0.089 (0.597)	0.68	(-1.752;0.922)
Frequentist	0.114	0.326	(-0.525;0.753)	0.696	−	(0;0.993)

Both the posterior mean and mode (in brackets) based on the draws from the posterior distribution are presented as parameter estimates of the MAREMA model; SD refers to the standard deviation of posterior draws; SE refers to the standard error; and 95% CI refers to the credibility interval in case of Bayesian estimation under the MAREMA model and confidence interval in case of the frequentist meta-analysis; no standard error is reported in the output of the metafor package for $\hat {\rho }$; the 95% CI of $\hat {\rho }$ in the frequentist meta-analysis was computed using the *Q*-profile method (Viechtbauer, 2007)

Parameter estimates of the MAREMA model and frequentist meta-analysis of the meta-analysis by Ho & Lee, (2012) were comparable. However, the estimates for *μ* were slightly larger under the MAREMA model relative to the frequentist meta-analysis estimate. Furthermore, as expected, the 95% credibility interval for *ρ* under the MAREMA model was considerably wider than the 95% confidence interval of the frequentist meta-analysis due to the fact that the random-effects model does not allow negative values for *ρ* and therefore there is less “room” for *ρ* to vary. To conclude, EMDR therapy was more efficacious than CBT therapy for treating PTSD, and heterogeneity was imprecisely estimated close to zero (indicating homogeneity).

Parameter estimates for *μ* under the MAREMA model were approximately zero whereas the frequentist meta-analytic estimate for *μ* was slightly larger for the meta-analysis by Whittaker et al.,, (2019). Furthermore, due to the skewness of the posterior of *ρ* under the MAREMA model there is a considerable difference between the posterior mean and posterior mode, where the latter is close to the estimate under the frequentist random-effects model. To conclude, estimates based on the MAREMA model and frequentist meta-analysis differed slightly for the meta-analysis of Whittaker et al.,, (2019). These difference were probably caused by the meta-analysis only containing three studies.

Given the uncertainty in the unconstrained estimates it is particularly useful to test precise null hypotheses on the overall effect *μ* and the relative heterogeneity *ρ*. Hence, Bayesian hypothesis testing under the MAREMA model is discussed next.

## Bayesian hypothesis testing under the MAREMA model

Testing hypotheses plays a fundamental role in scientific research in general and psychological science in particular. In this section, we propose multiple hypothesis tests for the mean *μ* and the relative between-study heterogeneity *ρ* separately.

We propose testing hypotheses for both *μ* and *ρ* under the MAREMA model. The following hypotheses are being tested for *μ*:

8 $$  \begin{aligned} H_{0}: \mu = 0 \\ H_{1}: \mu < 0 \\ H_{2}: \mu > 0, \end{aligned} $$

where support for *H*_0_, *H*_1_, or *H*_2_ indicates that the overall effect *μ* is equal to zero, is negative, or is positive, respectively. For *ρ* we test the following hypotheses:

9 $$  \begin{aligned} H_{0}: \rho = 0 \\ H_{1}: \rho < 0 \\ H_{2}: \rho > 0, \end{aligned} $$

where support for *H*_0_, *H*_2_, or *H*_1_ indicates a good fit of an equal-effect model, a random-effects model, or a model which assumes less variance due to sampling error (and thus a misfit of the equal-effect or random-effects model), respectively.

Bayes factors are used for testing the proposed hypotheses (Jeffreys, 1961; Kass & Raftery, 1995). A Bayes factor quantifies the evidence in the data for one hypothesis relative to a contrasting hypothesis via the ratio of the marginal likelihood,

10 $$ B_{12} = \frac{m_{1}(\mathbf{y})}{m_{2}(\mathbf{y})} $$

where **y** is a vector of the observed effect size *y*_*i*_ and *m*_1_ and *m*_2_ are the marginal likelihoods under *H*_1_ and *H*_2_, respectively. For example, *B*_12_ = 1 indicates that both hypotheses are equally supported by the data whereas *B*_12_ = 20 indicates that there is 20 times more support in the data for *H*_1_ relative to *H*_2_.

### Prior specification

In Bayesian hypothesis testing for the overall effect size, it is not recommended to use an arbitrarily vague prior to avoid Bartlett’s paradox (Jeffreys, 1961; Lindley, 1957; Bartlett, 1957). Instead, following (Mulder & Fox, 2019), we propose a unit-information prior for *μ* conditional on *ρ* in combination with a proper uniform prior for *ρ*. Under the unconstrained MAREMA model this boils down to

11 $$  \begin{aligned} \pi_{u}(\mu, \rho) & = \pi_{u} (\mu | \rho) \pi_{u}(\rho), \text{with} \\ \pi_{u}(\mu | \rho) & = N\Big(\mu, k \left( \mathbf{1}^{\prime} \mathbf{\Sigma}_{\rho}^{-1} \mathbf{1}\right)^{-1}\Big) \\ \pi(\rho) & = U(\rho_{min}, 1). \end{aligned} $$

The unit-information prior *π*(*μ*|*ρ*) contains the amount of information of a single study (Zellner, 1986). This is visible in the variance of *π*(*μ*|*ρ*) because the number of studies in a meta-analysis *k* is multiplied by the variance of $\hat {\mu }$, which is $(\mathbf {1}^{\prime } \mathbf {\Sigma }_{\rho }^{-1} \mathbf {1})^{-1}$ where **1** is a column vector of ones and $\mathbf {\Sigma }_{\rho } = \text {diag}\left ({{\sigma }_{1}^{2}}+\tau ^{2},...,{{\sigma }_{k}^{2}}+\tau ^{2}\right )$. Unit-information priors are commonly used for computing Bayes factors in model selection and hypothesis testing problems. For example, the well-known Bayesian information criterion (BIC, (Schwarz, 1978)) is based on an approximation of the marginal likelihood using a unit-information prior (Raftery, 1995; Kass & Wasserman, 1995). This class of priors is also employed in many other Bayesian testing scenarios (e.g., (Liang et al., 2008; Rouder & D Morey, 2012; Mulder et al., in press)). The usefulness of unit-information priors lies in the fact that they cover a reasonable range of possible values for the model parameters. As the prior contains the information of a single study, these priors are neither too informative nor too vague (to avoid Bartlett’s paradox). The prior *π*(*μ*|*ρ*) depends on *ρ* because *τ*^2^ is included in the variance-covariance matrix Σ_*ρ*_. Note that the prior *π*(*μ*|*ρ*) is different from the prior used for Bayesian estimation under the MAREMA model in Section “Bayesian estimation under the MAREMA model” as we used an improper prior for estimation whereas the prior *π*(*μ*|*ρ*) used for testing is a proper prior. The proper prior *π*(*ρ*) is the same prior distribution as we proposed for estimation under the MAREMA model.

**Fig. 2:**
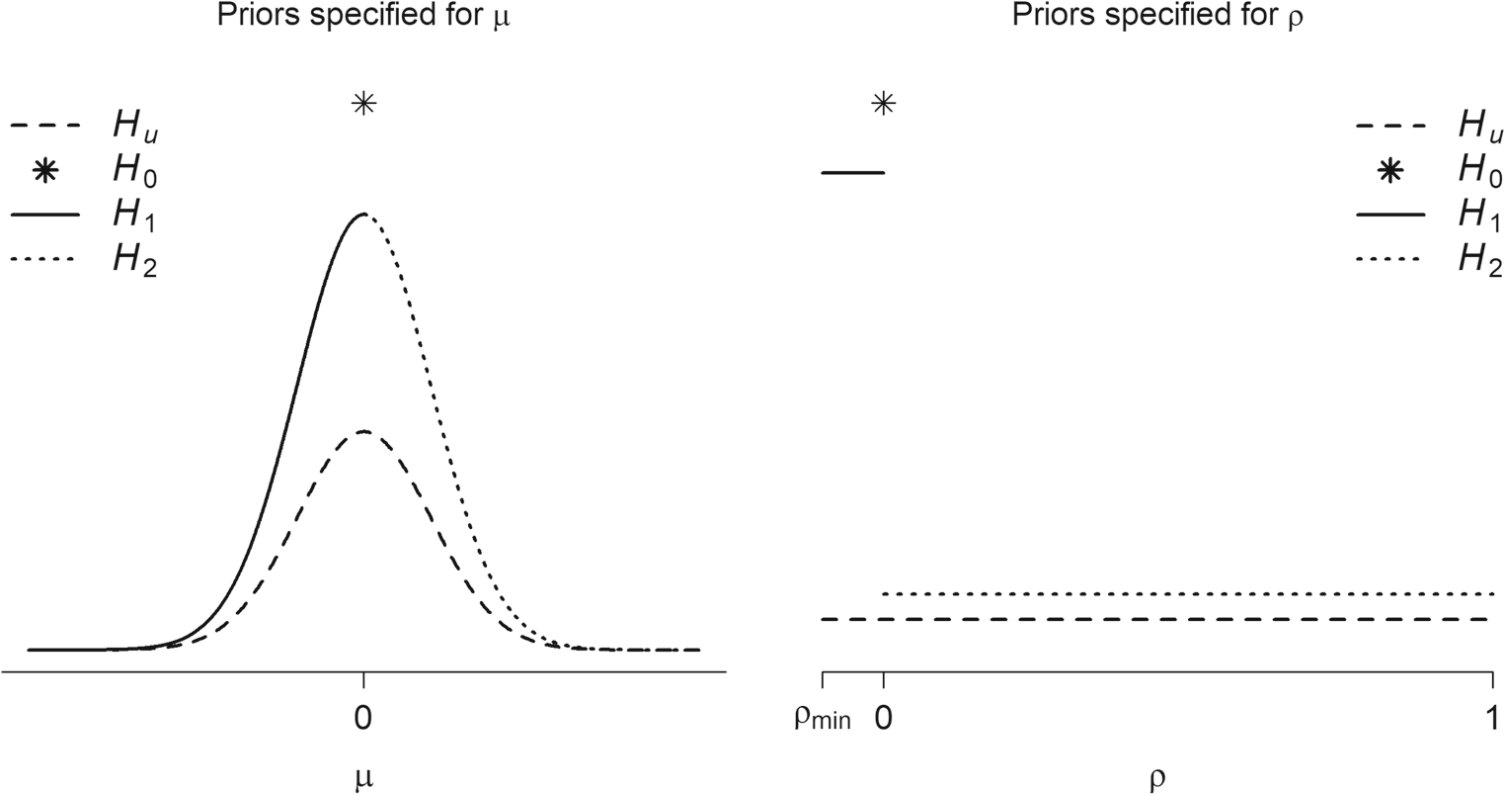
Prior distributions of *μ* (*left panel*) and *ρ* (*right panel*) for Bayesian hypothesis testing under the MAREMA model. The different prior distributions refer to the hypotheses listed in Eqs. 8 and 9

The unconstrained prior in Eq. 11 is used as building block for the priors under the hypotheses of interest. In particular, for the one-sided hypotheses, truncated priors are considered while the precise null hypotheses receive a point mass at the null value. Figure 2 illustrates the proposed prior distributions for testing unconstrained, one-sided, and point hypotheses under the MAREMA model. The left panel shows prior distributions for testing hypotheses regarding *μ* where *ρ* is left unconstrained. The dashed line refers to the prior distribution of the unconstrained hypothesis in Eq. 11. The asterisk at the top of the figure illustrates the point mass for testing the hypothesis *H*_0_ : *μ* = 0. The solid and dotted lines refer to the prior distributions for the one-sided hypotheses *H*_1_ : *μ* < 0 and *H*_2_ : *μ* > 0, respectively. Their heights are twice as large as the unconstrained prior to ensure that the distributions integrate to 1. The right panel shows prior distributions for testing hypotheses regarding *ρ*. The dashed, solid, and dotted lines refer to the prior distributions of the unconstrained (*H*_*u*_), left-sided (*H*_1_ : *ρ* < 0), and right-sided (*H*_2_ : *ρ* > 0) hypotheses whereas the asterisk refers to the point mass for the hypothesis of no between-study variance (*H*_0_ : *ρ* = 0).

### Marginal likelihood

The marginal likelihoods of the different hypotheses differ with respect to the prior distributions. Hence, the marginal likelihoods of the different hypotheses can be computed by using different prior distributions in combination with adjusting the limits of integration.

For example, the marginal likelihood for the one-sided hypothesis *H*_1_ : *μ* < 0 with *ρ* unconstrained can be written as a function of the marginal likelihood under the unconstrained model,

12 $$ m_{1}(\textbf{y}) = \iint_{\mu<0} f(\textbf{y}|\mu,\rho)\pi_{1}(\mu,\rho)d\mu d\rho = m_{u}(\textbf{y}) \frac{P(\mu < 0 | \mathbf{y}, H_{u})}{P(\mu < 0 | H_{u})},  $$

where *f*(**y**|*μ*,*ρ*) is the likelihood function of the MAREMA model in Eq. 5, *m*_*u*_(**y**) is the marginal likelihood under the unconstrained model, *P*(*μ* < 0|**y**,*H*_*u*_) and *P*(*μ* < 0|*H*_*u*_) are the posterior and prior model probabilities for *μ* < 0 under the hypothesis *H*_*u*_, and the prior under *H*_1_ can be written as a truncation of the unconstrained prior

$$ \pi_{1}(\mu,\rho) = \pi_{u}(\mu,\rho)I(\mu<0)/P(\mu < 0 | H_{u}), $$

where *I*(⋅) is the indicator function. Note here that $P(\mu < 0 | H_{u})=\frac {1}{2}$ because the unconstrained prior for *μ* is centered around 0. The posterior probability in Eq. 12 can be computed as the proportion of unconstrained posterior draws satisfying *μ* < 0. Interested readers are referred to Appendix B for further computational details.

## Software: BFpack

The R package BFpack (Mulder et al., in press) contains functions for computing Bayes factors for a large set of statistical models (e.g., multivariate regression, generalized linear models, and correlation analysis). As an argument the main function “BF” needs a fitted modeling object which defines under which model Bayes factors need to be computed. To execute the Bayes factor test under the MAREMA model using the “BF” function, the function needs as argument an object returned by fitting a random-effects meta-analysis model with the metafor package. The metafor package is popular software for conducting meta-analysis that can be used for any effect size measure and requires the meta-analyst to supply the observed effect sizes of the primary studies and the corresponding sampling variances (or standard errors). Hence, researchers familiar with the metafor package can readily compute the Bayes factors that we propose using the function BF. The “BF” function also returns unconstrained estimates of *μ* and *ρ* based on a Gibbs sampler.

## Bayesian hypothesis testing in examples

We compute Bayes factors using the MAREMA model for the two examples introduced in Section “?? ??”. The MAREMA model was fitted to the two meta-analyses using the proposed unit-information prior on *μ* and uniform prior on *ρ*.^1^ We tested the three hypotheses for *μ* and *ρ* listed in Eqs. 8 and 9, respectively. We analyzed the data using R (R Core Team, 2020) and the R packages metafor (Viechtbauer, 2010) and BFpack (Mulder et al., in press) in particular. R code illustrating how to compute Bayes factors and posterior probabilities for the hypotheses for the two examples is available at https://osf.io/ejfsv/.

The Bayes factors and posterior model probabilities for hypotheses on *μ* are presented in Table 2. For the meta-analysis by Ho & Lee, (2012), the Bayes factor comparing *H*_2_ with *H*_1_ is the largest of the tested hypotheses, which implies that the hypothesis *H*_2_ : *μ* > 0 is 15.810 times more likely than the hypothesis *H*_1_ : *μ* < 0. Moreover, the Bayes factor comparing *H*_2_ with *H*_0_ : *μ* = 0 equaled 3.779 implying that *μ* in this meta-analysis is likely larger than zero. Also the posterior probabilities (last row in Table 2; assuming equal prior probabilities) suggested that a positive effect (*H*_2_) was most likely after observing the data with a posterior probability of 0.753. The two-tailed frequentist hypothesis test of *H*_0_ : *μ* = 0 was not statistically significant using a significance level of 0.05 (*z* = 1.936, two-tailed *p* value= 0.053).

**Table 2:** Bayes factors and posterior model probabilities (*P*(*H*_*q*_|**y**)) for hypotheses on *μ*. The results based on the meta-analysis by Ho & Lee, (2012) are shown in the first columns and by Whittaker et al.,, (2019) in the last columns of the table

		Ho & Lee, (2012)			Whittaker et al.,, (2019)		
		*H*_0_	*H*_1_	*H*_2_	*H*_0_	*H*_1_	*H*_2_
	*H*_0_	1.000	4.183	0.265	1.000	2.558	2.115
Bayes factors	*H*_1_	0.239	1.000	0.063	0.391	1.000	0.827
	*H*_2_	3.779	15.810	1.000	0.473	1.209	1.000
*P*(*H*_*q*_|**y**)		0.199	0.048	0.753	0.537	0.210	0.254

*H*_0_ : *μ* = 0, *H*_1_ : *μ* < 0, and *H*_2_ : *μ* > 0

The Bayes factors and posterior model probabilities for hypotheses on *ρ* are shown in the first columns of Table 3 for the study by Ho & Lee, (2012). The Bayes factors comparing the hypotheses *H*_0_ : *ρ* = 0 with *H*_1_ : *ρ* < 0 and *H*_2_ : *ρ* > 0 indicated that *H*_0_ is approximately four and five times more likely, respectively. These results were also corroborated by the posterior probability that was the largest for *H*_0_ (with 0.689). This indicates that there was most evidence for an equal-effect model. The commonly used *Q*-test for testing whether the studies are homogeneous in a frequentist meta-analysis was not statistically significant (*Q*(9) = 9.417,*p* = 0.4). Note here that a nonsignificant result does not imply evidence for the null, as *p* values cannot be used for quantifying evidence for the null (because *p* values are by definition uniformly distributed if *H*_0_ would be true). To conclude, the EMDR treatment was observed to be on average more efficacious than the CBT treatment and effects are homogeneous across studies. However, due to the uncertainty in the posterior probabilities and the parameter estimates (see Section “?? ??”), more studies are required in order to draw more definite conclusions.

**Table 3:** Bayes factors and posterior model probabilities (*P*(*H*_*q*_|**y**)) for hypotheses on *ρ*. The results based on the meta-analysis by Ho & Lee, (2012) are shown in the first columns and by Whittaker et al.,, (2019) in the last columns of the table

		Ho & Lee, (2012)			Whittaker et al.,, (2019)		
		*H*_0_	*H*_1_	*H*_2_	*H*_0_	*H*_1_	*H*_2_
	*H*_0_	1.000	3.977	4.979	1.000	10.958	2.901
Bayes factors	*H*_1_	0.251	1.000	1.252	0.091	1.000	0.265
	*H*_2_	0.201	0.799	1.000	0.345	3.778	1.000
*P*(*H*_*q*_|**y**)		0.689	0.173	0.138	0.696	0.064	0.240

*H*_0_ : *ρ* = 0, *H*_1_ : *ρ* < 0, and *H*_2_ : *ρ* > 0

Bayes factors for testing hypotheses on *μ* for the meta-analysis by Whittaker et al.,, (2019) are shown in the last columns of Table 2. Hypothesis *H*_0_ : *μ* = 0 received more support than hypotheses *H*_1_ : *μ* < 0 and *H*_2_ : *μ* > 0, but there was no strong evidence for any of the hypotheses (largest Bayes factor equaled 2.558). This absence of strong evidence was likely also caused by this meta-analysis only consisting of three studies. The posterior model probabilities (last row Table 2) also showed that *H*_0_ was most likely (probability is 0.537). Application of a two-tailed frequentist hypothesis test resulted in a nonsignificant result, and thus *H*_0_ could not be rejected (*z* = 0.349, two-tailed *p* value= 0.727).

The hypothesis *H*_0_ : *ρ* = 0 received 10.958 more evidence than *H*_1_ : *ρ* < 0 and 2.901 more evidence than *H*_2_ : *ρ* > 0. Moreover, the hypothesis *H*_2_ : *ρ* > 0 was 3.778 more likely than *H*_1_ : *ρ* < 0. This corroborated the posterior model probabilities indicating that the effect sizes were most likely to be either homogeneous (with a probability 0.696) or heterogeneous (with a probability 0.240). Interestingly, the frequentist *Q*-test was statistically significant (*Q*(2) = 6.24,*p* = 0.044. This may be due to the fact that the significance tests rely on large sample theory, which may not be realistic here given there are only three studies. Using the posterior probabilities to get conditional error probabilities, there is a probability of 0.210 + 0.254 = 0.464 that we would be wrong when concluding that the hypothesis of no effect is true, and a probability of 0.064 + 0.240 = 0.304 that the hypothesis of homogeneous effects is true given the observed data. Thus (as expected) more data would be needed in order to receive more pronounced evidence about which hypothesis is likely to be true.

## Discussion

The main goals of meta-analyses are estimating the overall effect and the heterogeneity in effect size as well as drawing inferences for these parameters. This paper proposes novel Bayesian estimation and hypothesis testing methods to achieve these goals. Our approach is novel compared to alternative Bayesian meta-analysis methods because the framework builds on the MAREMA model where the (nuisance) study-specific effects are integrated out. This MAREMA model encompasses both an equal-effect and a random-effects meta-analytic model, and also encompasses a model which assumes that there is less variance than under the equal-effect and random-effects models.

Another major contribution is that we place a uniform prior distribution on the proportion of the variance that can be explained by between-study heterogeneity in true effect size rather than on the between-study variance or standard deviation in true effect size directly, as is usually done in Bayesian meta-analyses (Berry, 1998; Scheibehenne et al., 2017; Gronau et al., 2017). This relative variance has a clear standardized scale (Higgins et al., 2003) which facilitates its interpretation. Furthermore, the bounded parameter allows specifying a proper noninformative uniform prior in case prior information is absent or when a default (reference) test is preferred. Other advantages are that this prior does not depend on the effect size measure used in the meta-analysis, and avoids the need of eliciting a prior scale for which the Bayes factor can be highly sensitive.

We illustrated the proposed Bayesian hypothesis testing and estimation in two illustrative examples. Both the estimation and hypothesis testing results revealed large posterior uncertainty regarding the heterogeneity in true effect size. The uncertainty can be explained by the relatively small number of studies which is common in meta-analyses (Rhodes et al., 2015; Turner et al., 2015), and therefore the obtained quantifications for posterior uncertainty seemed reasonable. More convincing evidence for one of the tested hypotheses will be obtained if more studies become available and can be included in the meta-analysis. Note here that because Bayes factors are consistent the evidence in favor of the true hypothesis would go to infinity if the number of studies in a meta-analysis tends to infinity. The frequentist test for between-study heterogeneity on the other hand was statistically significant even though the example only included three studies. This may be another example that *p* values tend to overestimate the evidence against a null hypothesis (e.g., (Berger & Delampady, 1987; Sellke et al., 2001; Benjamin & Berger, 2019), and that Bayes factors and posterior probabilities may better capture the evidence in favor of or against statistical hypotheses in case of small samples.

In both applications, evidence was found that the heterogeneity (*ρ*) may be negative. This implies that an equal-effect meta-analysis model (where *ρ* = 0) or a random effects model (where *ρ* > 0) may not be appropriate, and might cause bias in the results. A negative *ρ* may also indicate a violation of the assumptions of the meta-analysis model, which is “corrected” by allowing *ρ* to be negative. For example, the reported within-study sampling variances ${\sigma _{i}^{2}}$ may be overestimated, the assumption of independent primary study’s effect sizes in a meta-analysis is violated, or the effect sizes of the primary studies were incorrectly computed (Ioannidis et al., 2006). More research is needed to explore the possible causes of a negative *ρ*, and how it affects the results. A good starting point would be (Nielsen et al., 2021) who explored the effect of negative intraclass correlations in a multilevel model.

We have only tested point hypotheses where the parameter was constrained to zero or one-sided hypotheses comparing hypotheses where the parameter was smaller or larger than zero. Other hypotheses that can be tested are combined hypotheses (e.g., *H* : *μ* > 0&*ρ* > 0) or hypotheses testing a parameter to another hypothesized value than zero. For example, meta-analysts may want to test point hypotheses using the rules of thumb for a small, medium, and large effect size as defined for many common effect size measures (for rules of thumb for multiple effect size measures see (Cohen, 1988)). Point hypotheses on the heterogeneity may be constrained to *ρ* = 0.25, 0.5, and 0.75 to resemble low, moderate, and high heterogeneity according to the thresholds proposed by (Higgins et al., 2003). It is important to note that any hypothesis on the proportion of total variance that can be explained by heterogeneity in true effect size can directly be transformed to a hypothesis on the between-study variance in true effect size. For example, testing the hypothesis *H* : *ρ* = 0 is equivalent to testing *H* : *τ*^2^ = 0. If a hypothesis on *ρ* is tested against another value than 0, the equivalent hypothesis on the between-study variance in true effect size can be obtained by transforming *ρ* to the between-study variance in true effect size.

We proposed a framework for Bayesian hypothesis testing and estimation using minimally informative default prior distributions, but our methodology can readily be extended to other prior distributions. Informative priors can be specified to incorporate external information about the heterogeneity in true effect size or overall effect from research in comparable fields. This may provide better estimates and statistical inferences especially if the number of primary studies in the meta-analysis is small. For example, if prior information about the heterogeneity parameter *ρ* is available this could be translated to an informative stretched beta prior distribution (following, (Mulder & Fox, 2019) for example) while prior information about the effect size could be translated to an informative normal prior. By considering different prior distributions one can assess the robustness of the quantification of the relative evidence in the data between statistical hypotheses under the MAREMA model.

Meta-analysts are generally not only interested in estimating and drawing inferences about the overall effect size and the between-study variance, but they are also interested in studying whether systematic differences between primary studies can explain the between-study variance. In case of large between-study variance, examining whether systematic differences between studies exist might even be more insightful than focusing on the overall effect size (Rouder et al., 2019). The between-study variance in a meta-analysis can be explained by including moderator variables in a meta-analysis model in a so-called meta-regression (Thompson & Sharp, 1999; Van Houwelingen et al., 2002). Future research may focus on extending our MAREMA model such that Bayesian estimation and Bayes factor testing can be conducted when moderators are included.

Future research may also focus on extending our methods to more advanced meta-analysis models such as multilevel meta-analysis (van den Noortgate & Onghena, 2003; Konstantopoulos, 2011) and multivariate meta-analysis (Jackson et al., 2011; Hedges, 2019). These models relax the strong assumption of the conventional random-effects model that effect sizes in a meta-analysis have to be independent. Another avenue for future research is studying whether relaxing the assumptions of the random-effects model benefit Bayesian meta-analysis under the MAREMA model in particular and Bayesian meta-analysis in general. For example, the random-effects model assumes that the within-study sampling variances are known (van Aert & Jackson, 2019; Jackson, 2009; Konstantopoulos & Hedges, 2019), and we adopted this assumption in the MAREMA model. This assumption is not tenable in practice which can be problematic if the sample size of studies is small. Estimates of the within-study sampling variance are then imprecise, which potentially lead to biased parameter estimates and inaccurate statistical inferences. This strong assumption can be avoided in a Bayesian meta-analysis by taking the uncertainty in these variances into account by means of a prior distribution instead of using a plug-in estimate. A logical choice for a prior distribution on the within-study sampling variances is the inverse-gamma distribution.

Another topic for future research is studying to what extent the proposed estimation and Bayes factor test under the MAREMA model are affected by publication bias. Publication bias implies that especially studies with statistically significant findings are more likely to be published than studies with non-significant findings. Consequently, studies with non-significant findings are more difficult to locate and are less likely to be included in a meta-analysis. Due to publication bias, effect sizes of primary studies and, in turn, also the overall effect size of a meta-analysis are most likely positively biased (Kraemer et al., 1998; van Assen et al., 2015; Lane & Dunlap, 1978). We expect estimation and inferences based on the MAREMA model to be inaccurate if publication bias is severe, and recommend researchers to also apply and report methods that correct for publication bias in this case.

To conclude, we have proposed Bayesian hypothesis testing and estimation using the MAREMA model. The proposed Bayes factors allow testing point and one-sided hypotheses for both the overall effect and heterogeneity in true effect size. We hope that our methods together with the easy-to-use software included in the R package BFpack enables researchers to frequently use Bayesian methods in their meta-analyses.

## Data Availability

All R code is available at https://osf.io/jcge7/ and https://osf.io/ejfsv/.

## Appendix A: Gibbs sampler for Bayesian estimation under the MAREMA model

We show in this appendix how the MAREMA model can be estimated by means of a Gibbs sampler. The likelihood of the MAREMA model given the prior distributions in Eq. 7 is

13 $$ \begin{array}{@{}rcl@{}} h(\mathbf{y} | \mu, \rho) &=& (2\pi)^{-\frac{k}{2}} \prod \left[ {\sigma}^{2}_{i} + \tilde{\sigma}^{2} \rho/(1-\rho) \right]^{-\frac{1}{2}}\\ && \times exp \left[ -\frac{1}{2} \sum \frac{(y_{i}-\mu)^{2}}{{\sigma}^{2}_{i}+\tilde{\sigma}^{2} \rho/(1 - \rho)} \right] (1 - \rho_{min})^{-1}. \end{array} $$

We first derive the conditional distribution of *μ*, i.e.,

14 $$ \begin{aligned} \mu | \mathbf{y}, \rho &\propto exp \left[ -\frac{1}{2} \sum \frac{(y_{i}-\mu)^{2}}{{\sigma}^{2}_{i}+\tilde{\sigma}^{2} \rho/(1-\rho)} \right] \\ &\propto exp \left[ -\frac{1}{2} \left( \mu^{2} \sum \frac{1}{ {\sigma}^{2}_{i}+\tilde{\sigma}^{2} \rho/(1-\rho)} - \mu \sum \frac{2 y_{i}}{ {\sigma}^{2}_{i}+\tilde{\sigma}^{2} \rho/(1-\rho)} \right) \right]. \end{aligned} $$

If we let $c = \sum 1/\left ({\sigma ^{2}_{i}}+\tilde {\sigma }^{2} \rho /(1-\rho )\right )$ and $v = \sum 2 y_{i}/({\sigma ^{2}_{i}}+\tilde {\sigma }^{2} \rho /(1-\rho ))$ and we complete the square for *μ*, we get

15 $$  \mu | \mathbf{y}, \rho \sim N \left( {}\frac{\sum y_{i} / ({\sigma}_{i}^{2}+\tilde{\sigma}^{2} \rho/(1-\rho))}{\sum 1 / ({\sigma}_{i}^{2}+\tilde{\sigma}^{2} \rho/(1-\rho))}, \frac{1}{\sum 1/({\sigma}^{2}_{i}+\tilde{\sigma}^{2} \rho/(1-\rho)} {}\right). $$

Using the conditional distribution of *μ* and a random walk procedure to approximate the conditional distribution of *ρ*, the Gibbs sampler can be written as

1. Set the initial value *ρ*_0_ and the variance of the proposal distribution *s*^2^.
2. Repeat the following steps to obtain *j* = 1, 2, ..., *J* draws and verify after every 100 draws whether the acceptance rate *a* in the random walk procedure is between 0.15 and 0.5 and adjust *s*^2^ to $\left [\sqrt {s^{2}} \left (\frac {a-0.5}{1-0.15}+1\right )\right ]^{2}$ if *a* > 0.5 and to $\left [\frac {\sqrt {s^{2}}}{2-a/0.15}\right ]^{2}$ if *a* < 0.15.
  - Draw a candidate sample *ρ*^∗^ from the proposal distribution *q*(*ρ*^∗^|*ρ*_*j*− 1_,*s*^2^), which is a truncated normal distribution with mean *ρ*_*j*− 1_, variance *s*^2^, lower bound of truncation $-\sigma ^{2}_{min}$, and upper bound of truncation 1.
  - Draw a *μ*_*j*_ from the conditional distribution of *μ* in Eq. 15 with *ρ* = *ρ*^∗^.
  - Compute the ratio
    $$ R = \frac{ h(\mathbf{y} | \mu_{j}, \rho^{*}) / q(\rho^{*} | \rho_{j-1}, s^{2})} { h(\mathbf{y} | \mu_{j}, \rho^{*}) / q(\rho_{j-1} | \rho^{*}, s^{2})}. $$
  - Draw a number from a uniform distribution ranging from 0 to 1 and denote this by *b*.
  - If *R* < *b*,*ρ*_*j*_ = *ρ*^∗^ otherwise *ρ*_*j*_ = *ρ*_*j*− 1_.
3. Exclude a burn-in period from the draws.

## Appendix B: Computation of marginal likelihoods

In this appendix, we describe how the marginal likelihoods of the hypotheses can be computed. Note that point and one-sided hypotheses are always specified for one parameter while the other parameter not included in hypothesis is left unconstrained.

### B.1: Computing the marginal likelihood of hypotheses with *μ* and *ρ* unconstrained (i.e., *H*_*u*_)

The marginal likelihood of the hypothesis where both *μ* and *ρ* are unconstrained is computed by first integrating out *μ* in *f*(**y**|*μ*, *ρ*)*π*(*μ*, *ρ*) (see Appendix C for the derivation),

16 $$ \begin{array}{@{}rcl@{}} m_{q}(\mathbf{y}){}&=&{}\int g(\mathbf{y} | \rho) d\rho = \int (2 \pi)^{-\frac{k+1}{2}} \prod \left[ {\sigma}^{2}_{i}+\tilde{\sigma}^{2} \rho/(1-\rho) \right]^{-\frac{1}{2}}\\ &&\times exp \left[ -\frac{1}{2} (\mathbf{y} - \hat{\mu} \mathbf{1})' \mathbf{\Sigma}_{\rho}^{-1} (\mathbf{y} - \hat{\mu} \mathbf{1}) \right] \left[ k (\mathbf{1}^{\prime} \mathbf{\Sigma}_{\rho}^{-1} \mathbf{1})^{-1} \right]^{-\frac{1}{2}} (1-\rho_{min})^{-1}\\ &&\times  exp \left[ -\frac{1}{2} (\mathbf{1}^{\prime} \mathbf{\Sigma}_{\rho}^{-1} \mathbf{1}) (\hat{\mu}^{2} - \frac{\hat{\mu}^{2}}{1+k^{-1}}) \right] (2\pi)^{\frac{1}{2}} (\mathbf{1}^{\prime} \mathbf{\Sigma}_{\rho}^{-1} \mathbf{1})^{-\frac{1}{2}} (1+k^{-1})^{-\frac{1}{2}} d\rho \end{array} $$

where *g*(**y**|*ρ*) is the likelihood where *μ* is integrated out and *q* refers to the particular hypothesis that is being tested. This marginal likelihood in Eq. 16 can then be approximated by using importance sampling,

17 $$ m_{u}(\mathbf{y}) d \rho = \int g(\mathbf{y} | \rho) \frac{\pi(\rho)}{w(\rho)} d \rho \approx J^{-1} \sum\limits_{j=1}^{J} g(\mathbf{y} | \rho) \frac{\pi(\rho)}{w(\rho_{j})} $$

where *w*(*ρ*) is a proposal density. This proposal density is a stretched beta distribution and was proposed by (Mulder & Fox, 2019) as a prior distribution for the ICC in hierarchical models. This stretched beta distribution ranges from *ρ*_*m**i**n*_ till 1. The shape parameters of this stretched beta distribution are computed with $\alpha = (\rho _{min}-\bar {\rho }) [(\bar {\rho }-1) \bar {\rho }-\bar {\rho }\rho _{min} + \rho _{min}+\lambda ]/[(\rho _{min}-1) \lambda ]$ and $\beta = \alpha (\bar {\rho }-1) / (\bar {\rho }-\rho _{min})$ where $\bar {\rho }$ and *λ* are the mean and variance of draws from the posterior distribution of *ρ*. Both shape parameters are multiplied by 0.6 to make sure that the proposal density has heavier tails than the posterior distribution. These heavier tails yield a more accurate approximation of the marginal likelihood.

### B.2: Computation of the marginal likelihood of *H*_0_ : *μ* = 0

The marginal likelihood of the point hypothesis *H*_0_ : *μ* = 0 has to be approximated because *ρ* could not be integrated out of *f*(**y**|*μ*,*ρ*)*π*(*μ*,*ρ*). Importance sampling is used for approximating this marginal likelihood,

18 $$ \begin{aligned} m_{0}(\mathbf{y}) & = \int I(\mu = 0) f(\boldsymbol{y} | \mu, \rho) d\rho \\ & = \int I(\mu = 0) f(\mathbf{y} | \mu, \rho) \frac{\pi(\rho)}{w(\rho)} d\rho \\ & \approx J^{-1} \sum\limits_{j=1}^{J} I(\mu = 0) f(\mathbf{y} | \mu, \rho) \frac{\pi(\rho)}{w(\rho_{j})} \end{aligned} $$

The proposal density *w*(*ρ*) is the stretched beta distribution that is also used for computing the marginal likelihood of hypothesis *H*_*u*_ (see Appendix B.1).

### B.3: Computation of the marginal likelihood of *H*_1_ : *μ* < 0

The computation of the marginal likelihood for the hypothesis *H*_1_ : *μ* < 0 is described in Section “Marginal likelihood”. The posterior model probability *P*(*μ* < 0|**y**, *H*_*u*_) is approximated using a random walk procedure that closely resembles the one in the Gibbs sampler in Appendix A and can be described as

1. Set the initial value *ρ*_0_ and the variance of the proposal distribution *s*^2^.
2. Repeat the following steps to obtain *j* = 1, 2, ..., *J* draws and verify after every 100 draws whether the acceptance rate *a* is between 0.15 and 0.5 and adjust *s*^2^ to $\left [\sqrt {s^{2}} \left (\frac {a-0.5}{1-0.15}+1\right )\right ]^{2}$ if *a* > 0.5 and to $\left [\frac {\sqrt {s^{2}}}{2-a/0.15}\right ]^{2}$ if *a* < 0.15.
  - Draw a candidate sample *ρ*^∗^ from the proposal distribution *q*(*ρ*^∗^|*ρ*_*j*− 1_,*s*^2^), which is a truncated normal distribution with mean *ρ*_*j*− 1_, variance *s*^2^, lower bound of truncation $-\sigma ^{2}_{min}$, and upper bound of truncation 1.
  - Compute the ratio
    $$ R = \frac{ g(\mathbf{y} | \rho^{*}) / q(\rho^{*} | \rho_{j-1}, s^{2})} { g(\mathbf{y} | \rho^{*}) / q(\rho_{j-1} | \rho^{*}, s^{2})}. $$
  - Draw a number from a uniform distribution ranging from 0 to 1 and denote this by *b*.
  - If *R* < *b*,*ρ*_*j*_ = *ρ*^∗^ otherwise *ρ*_*j*_ = *ρ*_*j*− 1_.
3. Draw samples from the conditional posterior of *μ* (denoted by *μ*_*j*_) given the sampled *ρ*_*j*_. The conditional posterior of *μ* is
  19 $$ N \left( \frac{\sum y_{i} / ({\sigma}^{2}_{i}+\tilde{\sigma}^{2} \rho_{j}/(1-\rho_{j})) + \mu_{0}/{\sigma}^{2}_{j}}{\sum 1 / ({\sigma}^{2}_{i}+\tilde{\sigma}^{2} \rho_{j}/(1-\rho_{j})) + 1/{\sigma}^{2}_{j}}, \frac{1}{\sum 1/({\sigma}^{2}_{i}+\tilde{\sigma}^{2} \rho_{j}/(1-\rho_{j})) + 1/{\sigma}^{2}_{j}} \right) $$
  where *μ*_0_ = 0 and ${\sigma ^{2}_{j}} = \frac {k}{\sum 1/({\sigma ^{2}_{i}}+\tilde {\sigma }^{2} \rho _{j}/(1-\rho _{j}))}$.
4. Exclude a burn-in period from the draws.
5. Compute how many draws of *μ*_*j*_ are smaller than 0 to approximate *P*(*μ* < 0|**y**, *H*_*u*_).

### B.4: Computation of the marginal likelihood of *H*_2_ : *μ* > 0

Computation of the marginal likelihood of the hypothesis *H*_2_ : *μ* > 0 is highly similar to that of *H*_1_ : *μ* < 0. That is, the marginal likelihood can be approximated with

20 $$ m_{1}(\mathbf{y}) = \iint_{\mu>0} f(\boldsymbol{y} | \mu, \rho) \pi_{2}(\mu, \rho) d\mu d\rho = m_{u}(\mathbf{y}) \frac{P(\mu > 0 | \mathbf{y}, H_{u})}{P(\mu > 0 | H_{u})} $$

where *P*(*μ* > 0|**y**,*H*_*u*_) and *P*(*μ* > 0|*H*_*u*_) are the posterior and prior model probabilities for *μ* > 0 under the hypothesis *H*_*u*_ where both *μ* and *ρ* are unconstrained. The probability *P*(*μ* > 0|*H*_*u*_) is computed using the prior distribution *π*(*μ*|*ρ*). The posterior model probability *P*(*μ* > 0|**y**,*H*_*u*_) is approximated by means of the random walk procedure described in Appendix B.3 where is computed how many draws are larger instead of smaller than 0 in the final step.

### B.5: Computation of the marginal likelihood of *H*_0_ : *ρ* = 0

The marginal likelihood of the hypothesis *H*_0_ : *ρ* = 0 can be computed with the likelihood function *g*(**y**|*ρ*) in Eq. 16 where *μ* is integrated out,

21 $$ m_{0}(\mathbf{y}) = I(\rho = 0) g(\mathbf{y} | \rho). $$

### B.6: Computation of the marginal likelihood of *H*_1_ : *ρ* < 0

The marginal likelihood of the hypothesis *H*_1_ : *ρ* < 0 is computed in a similar way as the marginal likelihood for one-sided hypotheses on *μ*. That is,

22 $$ m_{1}(\mathbf{y}) = {\int}_{\rho < 0} g(\mathbf{y} | \rho) d\rho = m_{0}(\mathbf{y}) \frac{P(\rho < 0 | \mathbf{y}, H_{u})}{P(\rho < 0 | H_{u})} $$

where *P*(*ρ* < 0|**y**,*H*_*u*_) and *P*(*ρ* < 0|*H*_*u*_) are the posterior and prior model probabilities for *ρ* < 0 under the hypothesis *H*_*u*_ where *μ* and *ρ* are unconstrained. The probability *P*(*ρ* < 0|*H*_*u*_) is computed using the prior *π*(*ρ*). The probability *P*(*ρ* < 0|**y**,*H*_*u*_) is approximated by means of the random walk procedure described in Appendix B.3 by computing how many draws of *ρ*_*j*_ are smaller than 0.

### B.7: Computation of the marginal likelihood of *H*_2_ : *ρ* > 0

Computation of the marginal likelihood of the hypothesis *H*_2_ : *ρ* > 0 is highly equivalent to that of *H*_1_ : *ρ* < 0,

23 $$ m_{2}(\mathbf{y}) = {\int}_{\rho > 0} g(\mathbf{y} | \rho) d\rho = m_{0}(\mathbf{y}) \frac{P(\rho > 0 | \mathbf{y}, H_{u})}{P(\rho > 0 | H_{u})} $$

with the exception that *P*(*ρ* > 0|**y**,*H*_*u*_) and *P*(*ρ* > 0|*H*_*u*_) are now the posterior and prior model probability of *ρ* > 0. The posterior model probability *P*(*ρ* > 0|**y**,*H*_*u*_) is approximated by computing how many draws of *ρ*_*j*_ are larger than 0 in the random walk procedure described in Appendix B.3.

## Appendix C: Derivation of marginal likelihood of MAREMA model where *μ* is integrated out

We show in this appendix how the marginal likelihood in Eq. 16 can be obtained by integrating out *μ* in *f*(**y**|*μ*, *ρ*)*π* (*μ*, *ρ*). The marginal likelihood of the MAREMA model in Eq. 16 equals

24 $$ \begin{array}{@{}rcl@{}} &&\int \int f(\boldsymbol{y} | \mu, \rho) d\mu d\rho = \int \int (2\pi)^{-\frac{k}{2}} \prod \left[ {\sigma}_{i}^{2} + \tilde{\sigma}^{2} \rho/(1-\rho) \right]^{-\frac{1}{2}}\\ &&\times exp \left[{}-\frac{1}{2} \sum \frac{(y_{i}-\mu)^{2}}{{\sigma}^{2}_{i}+\tilde{\sigma}^{2} \rho/(1-\rho)} {}\right] (2\pi)^{-\frac{1}{2}} \left[ {}\frac{k}{\sum \left( {\sigma}^{2}_{i}+\tilde{\sigma}^{2} \rho/(1-\rho)\right)^{-1}} {}\right]^{-\frac{1}{2}} \\ &&\times exp \left[ -\frac{1}{2} \frac{\mu^{2}}{k/\sum ({\sigma}^{2}_{i}+\tilde{\sigma}^{2} \rho/(1-\rho))^{-1}} \right] (1-\rho_{min})^{-1} d\mu d\rho. \end{array} $$

Combining powers and replacing some expressions with their equivalents in matrix algebra yields

25 $$ \begin{array}{@{}rcl@{}} \int \int f(\boldsymbol{y} | \mu, \rho) d\mu d\rho= \int \int (2\pi)^{-\frac{k+1}{2}} \prod \left[ {\sigma}^{2}_{i} + \tilde{\sigma}^{2} \rho/(1-\rho) \right]^{-\frac{1}{2}} \\ \times exp \left[ -\frac{1}{2} (\mathbf{y}-\hat{\mu} \mathbf{1})' \mathbf{\Sigma}_{\rho}^{-1} (\mathbf{y}-\hat{\mu} \mathbf{1}) \right] \left[ k (\mathbf{1}^{\prime} \mathbf{\Sigma}_{\rho}^{-1} \mathbf{1})^{-1} \right]^{-\frac{1}{2}} (1-\rho_{min})^{-1} \\ \times exp \left[ -\frac{1}{2} \left( \frac{(\mu-\hat{\mu})^{2}}{(\mathbf{1}^{\prime} \mathbf{\Sigma}_{\rho}^{-1} \mathbf{1})^{-1}} + \frac{k^{-1} \mu^{2}}{(\mathbf{1}^{\prime} \mathbf{\Sigma}_{\rho}^{-1} \mathbf{1})^{-1}} \right) \right] d\mu d\rho. \end{array} $$

Rewriting Eq. 25 to facilitate integrating out *μ* gives

26 $$ \begin{array}{@{}rcl@{}} &&{}\int \int f(\boldsymbol{y} | \mu, \rho) d\mu d\rho= \int \int (2\pi)^{-\frac{k+1}{2}} \prod \left[ {\sigma^{2}_{i}} + \tilde{\sigma}^{2} \rho/(1-\rho) \right]^{-\frac{1}{2}}\\ &&\times exp{\kern-1.5pt} \left[ -\frac{1}{2} (\mathbf{y}-\hat{\mu} \mathbf{1})' \mathbf{\Sigma}_{\rho}^{-1} (\mathbf{y}-\hat{\mu} \mathbf{1}) \right] \left[ k (\mathbf{1}^{\prime} \mathbf{\Sigma}_{\rho}^{-1} \mathbf{1})^{-1} \right]^{-\frac{1}{2}} (1-\rho_{min})^{-1} \\ &&\times exp{\kern-1.5pt} \left[ {}-\frac{1}{2} \frac{(\mu-\hat{\mu}/(1+k^{-1}))^{2}}{(\mathbf{1}^{\prime} \mathbf{\Sigma}_{\rho}^{-1} \mathbf{1})^{-1} (1+k^{-1})^{-1}} {}\right] exp {\kern-1.5pt}\left[{} - \frac{1}{2} (\mathbf{1}^{\prime} \mathbf{\Sigma}_{\rho}^{-1} \mathbf{1}) (\hat{\mu}^{2}-\hat{\mu}^{2}/(1+k^{-1}) {}\right] d\mu d\rho. \end{array} $$

Integrating out *μ* of Eq. 26 yields the marginal likelihood in Eq. 16.

## Footnotes

1 The random walk procedures were based on 100,000 iterations (burn-in period of 5000 iterations) and starting values *ρ*_0_ equal to the estimate of a frequentist random-effects meta-analysis with the restricted maximum likelihood as estimator for the between-study variance and $s^{2} = \sqrt {0.1}$
